# Microneedle Patches with O_2_ Propellant for Deeply and Fast Delivering Photosensitizers: Towards Improved Photodynamic Therapy

**DOI:** 10.1002/advs.202202591

**Published:** 2022-07-15

**Authors:** Pei Liu, Yangxue Fu, Fulong Wei, Teng Ma, Jingli Ren, Zhanjun Xie, Shanzheng Wang, Jinjin Zhu, Lianbin Zhang, Juan Tao, Jintao Zhu

**Affiliations:** ^1^ Hubei Engineering Research Center for Biomaterials and Medical Protective Materials and State Key Laboratory of Materials Processing and Mold Technology School of Chemistry and Chemical Engineering Huazhong University of Science and Technology (HUST) Wuhan 430074 China; ^2^ Department of Dermatology Union Hospital Tongji Medical College HUST Wuhan 430022 China; ^3^ School of Energy and Power Engineering HUST Wuhan 430074 China

**Keywords:** gaseous oxygen propellant, improved transdermal delivery, microneedle patches, photodynamic therapy, tumor treatment

## Abstract

Photodynamic therapy (PDT) is an emerging technique for treating tumors. Especially, topical administration of photosensitizers (PSs) is more favorable for superficial tumor treatments with low systematic phototoxicity. Yet, ineffective migration of PSs to targeted tumor tissues and rapid consumption of O_2_ during PDT greatly limit their effects. Herein, PS‐loaded microneedle (MN) patches with O_2_ propellant for a deeper and faster transdermal delivery of PS and improved PDT by embedding sodium percarbonate (SPC) into dissolving poly(vinyl pyrrolidone) MNs are presented. It is shown that SPC in the MNs can react with surrounding fluid to generate gaseous oxygen bubbles, forming vigorous fluid flows and thus greatly enhancing PS of chlorin e6 (Ce6) penetration in both hydrogel models and skin tissues. Reactive oxygen species (ROS) in hypoxic breast cancer cells (4T1 cells) are greatly increased by rapid penetration of PS and relief of hypoxia in vitro, and Ce6‐loaded SPC MNs show an excellent cell‐killing effect. Moreover, lower tumor growth rate and tumor mass after a 20‐d treatment in tumor‐bearing mice model verify the improved PDT in gaseous oxygen‐droved delivery of PS. This study demonstrates a facile yet effective route of MN delivery of PSs for improved PDT in hypoxic tumor treatment.

## Graphical abstract

Photosensitizer‐loaded microneedles with O_2_ propellant that can actively deliver photosensitizer to the tumor by generating gaseous oxygen bubbles are developed, greatly enhancing the penetration of photosensitizer (PS) and relieving the hypoxia for improved photodynamic therapy.

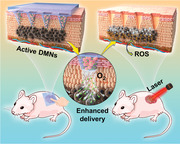

## 1 Introduction

Photodynamic therapy (PDT) has become a promising and efficient strategy for the treatment of various tumors and non‐neoplastic diseases due to its noninvasive nature and spatiotemporal selectivity.^[^ ^1^ ^]^ In a typical PDT, the exogenous photosensitizers (PSs) undergo a series of photochemical reactions with oxygen under the light of a specific wavelength to generate high cytotoxic reactive oxygen species (ROS), thereby selectively destroying the target cells or tissues with efficient outcomes, minimal side effects, and nondrug resistances.^[^ ^2^ ^]^ PDT has been widely applied in the clinical local treatment of various cancers, especially superficial tumors considering the limited penetration depth of the light irradiation.^[^ ^3^ ^]^ In general, the successful implementation of the PDT for the tumor treatment replies on the delivery and the accumulation of PS in the targeted tumor tissues.^[^ ^4^ ^]^ Especially, the transdermal delivery of the PS to the targeted sites holds great promise in tumor treatments due to the low systematic toxicity.^[^ ^5^ ^]^

Recently, many techniques have been explored to further increase the transdermal delivery of PSs, including the help of penetration enhancers,^[^ ^6^ ^]^ nanoemulsions,^[^ ^7^ ^]^ iontophoresis,^[^ ^8^ ^]^ and microneedle (MN)‐based patches delivery.^[^ ^9^ ^]^ Among these, MN patches have received widespread attention in transdermal drug delivery due to their advantages of painlessness, minimal invasion, uniform drug delivery, safety, and high efficiency.^[^ ^10^ ^]^ Although dissolving MNs show good PDT effects for tumors,^[^ ^11^ ^]^ the traditionally tapered MNs usually show imperfect skin insertion due to the viscoelasticity of the skins,^[^ ^12^ ^]^ which would significantly reduce its delivery effectiveness.^[^ ^13^ ^]^ Besides, the passive diffusion of payloads in the skin also limits the penetration depth of the loaded PSs.^[^ ^14^ ^]^ Therefore, a significantly prolonged treating duration and increased amount of PSs are usually required to achieve the therapeutical effect. On the other hand, the abnormal metabolism and rapid growth of the tumor cells may consume a lot of oxygen, and disordered tumor blood vessels cannot supply sufficient oxygen which is essential for the PDT process.^[^ ^15^ ^]^ As a result, the partial pressure of oxygen at the tumor site is usually lower than that at normal tissues, and the hypoxic environment will severely inhibit the effect of PDT.^[^ ^16^ ^]^ Therefore, an all‐in‐one MN delivery platform that combines the characteristics for both active delivery and hypoxia relief is highly desirable but has been never explored.

Herein, we present PS‐loaded dissolving MN patches with O_2_ propellant (PS‐loaded active DMNs) that could actively deliver PS to the tumor site by generating gaseous oxygen bubbles, greatly enhancing the penetration depth and the rate of PS and relieving the hypoxia of the tumor during the laser irradiation for improved PDT. The PS‐loaded active DMNs are made of a dissolving polymer of polyvinyl pyrrolidone (PVP) loaded with PS of chlorin e6 (Ce6) and sodium percarbonate (SPC) particles, which could effectively deliver PS to the tumor with high targeting efficiency. Upon skin insertion, the MNs dissolved immediately, and the encapsulated SPC particles can instantaneously react with the dermal interstitial fluid (ISF) in the surrounding environment and generate oxygen bubbles, producing a violent vortex in the ISF and powering up the penetration of the PSs. The improved release and migration of PS were verified by both theoretical simulation and experimental observation in vitro by recording the movement of fluorescence signal in water, Pluronic F‐127 gel, and pigskin model, and a greatly enhanced penetration depth was demonstrated via active delivery of SPC MNs. Different delivery behaviors were verified in hypoxic breast cancer cells (4T1 cells) and tumor‐bearing mice. Due to the enhanced penetration of PS driven by oxygen bubbles and relief of tumor hypoxia, the cell‐killing effect can be significantly increased from 29.2% to 99.0%. In vivo experiments in tumor‐bearing mice models demonstrated that tumor growth was significantly inhibited, and the survival rate increased 50% by the active delivery. The current study provides an efficient and rapid delivery of PSs, and the generated gaseous oxygen could relieve the tumor hypoxia, providing a new strategy for a high‐efficient PDT effect.

## 2 Results and Discussion

**Figure 1:**
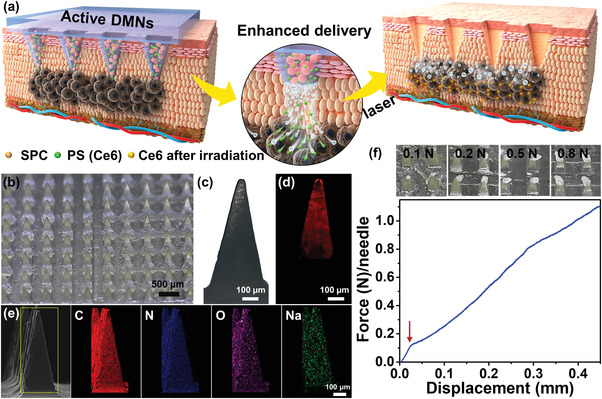
Ce6‐loaded active DMNs for enhanced transdermal drug delivery. a) Illustration showing the Ce6‐loaded active DMNs for enhanced PS delivery, SPC reacts with dermal interstitial fluid to generate gaseous oxygen bubbles, served as a pump for promoting the penetration of PS to the deep tumor. b–f) Fabrication and characterization of Ce6‐loaded active DMNs: (b) Optical images; (c,d) representative optical microscopy images of a single needle: (c) bright field, (d) fluorescence field. (e) The EDX analysis of the Ce6‐loaded active DMNs, scale bar in the last image applies to the others. (f) Compression curve of the MNs and the corresponding optical microscopy images with different applied forces.

The PS‐loaded active DMNs (**Figure** **1**a) can be readily fabricated by a micro‐molding method as schematically illustrated in Figure S1a (Supporting Information), which consists of SPC particles (Figure S1b, Supporting Information), PS of Ce6, and PVP. PVP was chosen as the polymer matrix for its hydrophilicity and biocompatibility. SPC, as a chemical oxygen source for emergency oxygen, can generate gaseous oxygen when being in contact with water.^[^ ^17^ ^]^ The generated oxygen bubbles can form convection at the localized site for enhancing penetration of PS to the deep tumor and meanwhile relieve tumor hypoxia transitorily. As shown in Figure 1b, the MN patch showed a 10×10 MN array with the base diameter ≈ 300 µm, height ≈ 800 µm, and center‐to‐center space ≈ 750 µm, respectively. A fluorescent microscopy image of a single shaft showed that Ce6 (red signal) was mainly distributed in the MN tip with bright and fluorescence microscopy images (Figure 1c,d). Scanning electron microscopy (SEM) images of a single Ce6‐loaded active DMN tip and energy dispersive X‐ray spectroscopy (EDX) mapping analysis clearly showed that the microparticles were uniformly dispersed in the polymer matrix (Figure 1e). The loading amount of Ce6 was evaluated by dissolving the MNs containing Ce6 in the water, followed by UV–vis spectrum measurement, and the result revealed that the loading capacity was 2.96 ± 0.33 µg per patch. The mechanical strength of Ce6‐loaded active DMNs made with the SPC content of 42.9% was measured under compression, and the results showed that the breaking point of each needle was 0.11 ± 0.09 N, which is higher than the reported minimum effective force required to pierce the skin (0.045 N per needle),^[^ ^18^ ^]^ thus confirming its potential ability to pierce the skin and its scalability for in vivo applications. According to the ratio of the destructive force to the minimum force for MN insertion, the safety factor of our current MNs is calculated to be 2.44, higher than the minimum safety factor of 2,^[^ ^19^ ^]^ ensuring the integrality of MNs during the insertion process. Besides, the MN tip was observed through an optical microscope, and it could be seen that the tip structure of the MN was damaged after applying loads > 0.1 N per needle (Figure 1f).

We then studied the dissolution and gaseous oxygen generation of the active DMNs with different loading amounts of SPC (i.e., 9.1%, 20%, 33.3%, and 42.9%) to optimize the gas generation of the MN patches. It was found that once the MN patch contacted with phosphate buffer (PBS) solution, the MN shaft quickly dissolved and exposed the SPC particles to the surrounding fluid, producing gaseous oxygen bubbles rapidly (Figure S2a, Supporting Information). The gaseous oxygen was evaluated from different patches with varied SPC contents. As expected, the amount of oxygen generated increased with the loaded SPC, with the MN patch of SPC content being 42.9% producing gaseous O_2_ in PBS solution of 3.98 ± 0.08 mg per (L patch) (Figure S2b, Supporting Information). Considering the preservation of the patches, loading amount of 42.9% for the SPC was chosen for the preparation of the active DMNs. Besides, we found that the dissolving and O_2_ generation of the active DMNs may accelerate with the temperature raise from 25 to 37 °C (Figure S2c, Supporting Information), which promised the O_2_ generation of the active DMNs in vivo.

**Figure 2:**
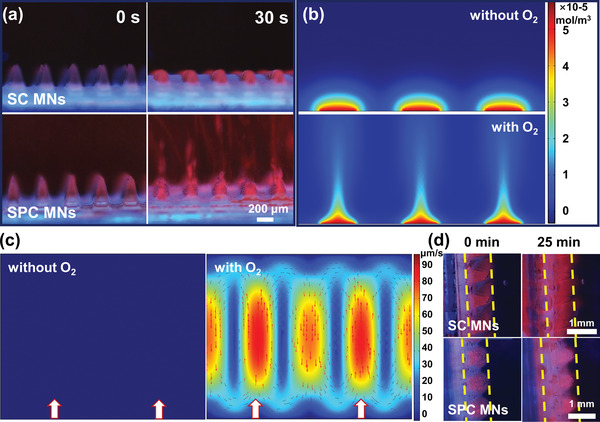
Evaluation of the in vitro payload release performance of Ce6‐loaded active SPC MNs and Ce6‐loaded passive SC MNs. a) The dissolution state of Ce6‐loaded passive SC MNs and Ce6‐loaded active SPC MNs in PBS at 0 and 30 s. COMSOL Multiphysics simulation of b) the motion of Ce6 and c) the water flow generated by the absence and presence of O_2_, the white arrow presents the location of O_2_. d) Time‐lapse fluorescence image (side view) of MNs inserted into a 2‐mm‐thick F‐127 gel at 0 and 25 min. Scale bar in the last image applies to the others in (a) and (d).

The introduction of the SPC into the MNs and the thus‐resulted gaseous oxygen greatly prompted the dissolution of the MNs and the migration of the incorporated payloads. First, we monitored the dissolving process of the Ce6‐loaded active DMNs in PBS solution by fluorescent microscopic imaging. As a comparison, passive dissolving MNs were also prepared with sodium carbonate (SC) replacing the SPC. We found that once being in contact with the PBS solution, the polymeric matrix quickly dissolved with clearly observable gas bubbles within 10 s (Video S1, Supporting Information), which accordingly prompted the diffusion of the payload of Ce6 as evidenced by the fluorescent microscopy images in **Figure** **2**a. It was observed that the Ce6 migrated 1 mm within 30 s, while in the MN patch with SC, the Ce6 released with the dissolution of PVP but no obvious migration could be observed at 30 s (Video S2, Supporting Information). To further verify the prompted migration of the Ce6, we employed COMSOL Multiphysics simulation by transient laminar flow model to simulate the flow process in the water. The simulation results showed that the Ce6 remained around the patch with passive diffusion without O_2_ (Figure 2b). In sharp contrast, bubbles rose under the action of buoyancy, which accelerated the moving speed of the water; therefore, strong convection (maximum flow rate of 90 µm s^−1^) can be formed with the O_2_ pressure, which accelerated the migration of Ce6 (Figure 2c). Consistent with the experimental results, this simulation indicates that the addition of SPC could promote the migration of Ce6 loaded in MNs. Then, we evaluated the potential of active DMNs on promoting penetration of the payload in the hydrogel model. The longitudinal permeability of Ce6‐loaded active SPC MNs was assessed by measuring the migration distance of the red fluorescent signal in the transparent F‐127 hydrogel, which has been widely used for simulating the skin tissues.^[^ ^20^ ^]^ Fluorescent microscopy images of side view were taken at different intervals (5 min) and the results (0 and 25 min) confirmed that the significantly accelerated diffusion of Ce6 was observed, showing a larger penetration depth (i.e., 918 ±12 µm for SC MNs vs 1130 ± 65 µm for SPC MNs) due to the gaseous oxygen bubbles generated by SPC particles (Figure 2d). These results demonstrated that the migration speed of the payload increased in all directions under the O_2_ pressure. In addition, the release of passive and active MNs was investigated by using pig cadaver skin, and the penetration depth was plotted as a function of time (Figure S3a,b, Supporting Information). The results showed that the improved permeability and faster transdermal transport of payload were also significantly pronounced in isolated pigskin tissue by active DMNs.

**Figure 3:**
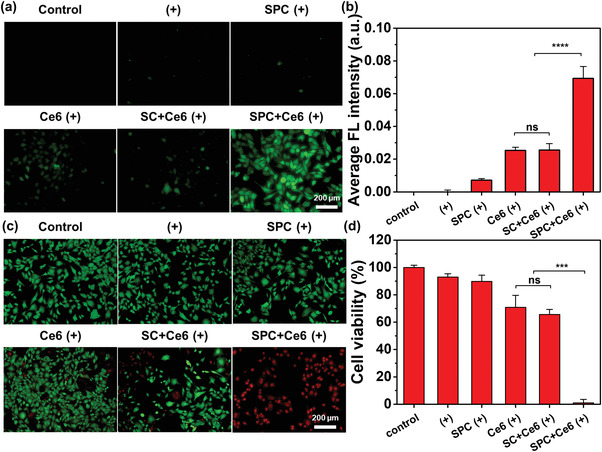
In vitro PDT of Ce6‐loaded active DMNs (1 patch mL^−1^). a) Fluorescence microscopy images of hypoxic 4T1 cells after different treatments and stained by DCFH‐DA probe: no treatment (control), laser (+), SPC (+), Ce6 (+), SC+Ce6 (+), and SPC+Ce6 (+). b) Corresponding average fluorescence intensity (FL intensity) of hypoxic 4T1 after different treatments. c,d) Cell‐killing effect of hypoxic 4T1 cells after different treatments: Calcein‐AM/PI staining (c) and cell viability (d). The scale bar in the last image applies to the others in (a) and (c). The results in (b) and (d) were shown as the mean ± SD. Statistical significance was calculated by student's *T*‐test (*n* ≥ 4). ns, *p* > 0.05, *p < 0.05, **p < 0.01, ***p < 0.001, ****p < 0.0001.

Besides the prompted delivery of the PS, the gaseous oxygen also improved the PDT effect. Taking hypoxia (1% O_2_) breast cancer cells (4T1) as models, we first evaluated the intracellular ROS level by using a 2,7‐dichlorofluorescin diacetate (DCFH‐DA) probe. The DCFH‐DA was non‐fluorescent, it could be hydrolyzed by esterase and followed by being oxidized by ROS in cell to form a compound with strong green fluorescence signal. Compared to the SC group, we found that the addition of SPC could increase the average fluorescence intensity of the probe that reflects the intracellular ROS level, by 2.37 times, suggesting that a more efficient cell‐killing effect was achieved (Figure S4a,b, Supporting Information). Notably, the dissolution of SPC did not lead to the pH change of PBS solution (Figure S5a, Supporting Information) and obvious cell‐killing to hypoxic 4T1 cells or 3T3 cells when the concentration of SPC was lower than 100 µg mL^−1^ (Figure S5b,c, Supporting Information). Furthermore, we explored the influence of Ce6‐loaded active DMNs on the enhancement of intracellular ROS in agarose hydrogel‐covered hypoxic 4T1 cells. The hypoxic 4T1 cells were divided into six groups: no treatment (control group); laser group (+); SPC MNs with laser irradiation (SPC (+)); Ce6‐loaded MNs with laser irradiation (Ce6 (+)); Ce6‐loaded SC MNs with laser irradiation (SC+Ce6 (+)); Ce6‐loaded SPC MNs with laser irradiation (SPC+Ce6 (+)). The MN patch was inserted into the phantom‐mimicking tissue prepared by agarose covered on hypoxia 4T1 cells, and the intracellular ROS and cell viability were tested after being irradiated with 655 nm laser (0.1 W cm^−2^, 3 min). Without Ce6, the green fluorescence signal of probe in the SPC (+) group had a slight increase compared with that in the laser group, which could be attributed to the relief of hypoxia by gaseous oxygen released from active DMNs. Once the Ce6 was added, the ROS in hypoxic 4T1 cells was significantly increased under sufficient PS, especially with increased gaseous oxygen from Ce6‐loaded active DMNs, which could be attributed to the enhanced penetration and the relief of hypoxia (**Figure** **3**a,b). Correspondingly, the qualitative and quantitative analysis of the cell‐killing effect illustrated the improved PDT in hypoxic cells by Ce6‐loaded active DMNs. Compared to the control group, laser irradiation cannot kill the 4T1 cells effectively even with the SPC. While the Ce6 was loaded in the MNs, the cell viability was significantly reduced to 70.8%, indicating that Ce6 showed good PDT after laser irradiation (Figure 3c,d). With the addition of oxygen generated by SPC, due to the relief of hypoxia and rapid migration of Ce6 in a short incubation time (30 min), the cell viability decreased rapidly to 1.0% after laser irradiation. While without laser irradiation, there was no change in ROS level (Figure S6, Supporting Information) and cell viability (Figure S7, Supporting Information), indicating that the weak oxidation property of SPC at low concentration had no obvious cytotoxicity. All the results suggest that SPC simultaneously promoted the downward migration of the PS and improved the hypoxic microenvironment of tumor cells.

Moreover, we investigated the skin insertion ability and biocompatibility in vivo. The MNs were pressed onto the dorsal skin of hair‐removed mice by thumb and kept 5 min for a complete dissolution of MNs tips (Figure S8a, Supporting Information). After removing the MNs, an obvious array of micropores were left on the skin, and the recovery process was continuously recorded through an optical microscope to evaluate the compatibility of the active SPC MNs (Figure S8b, Supporting Information). The puncture marks gradually became invisible, and the spots on the skin disappeared after 1 h, and the skin recovered after 24 h. The histological section of dorsal mice skin with a needle‐like channel ≈ 200 µm at the puncture site showed that the Ce6‐loaded DMNs successfully pierced the normal epidermis (Figure S8c, Supporting Information). The distance between the upper edge of the tumor and the skin surface was 0.37 ± 0.04 mm (Figure S8d, Supporting Information); therefore, the MNs did not contact with tumor during the insertion process, which would avoid the tumor metastasis caused by microchannels created by MN penetration. Furthermore, the cell viability of 3T3 remained > 90.1% with the Ce6‐loaded MNs treatments (Figure S8e, Supporting Information), and there was no obvious increase of IL‐1*β* on the immunohistochemical staining analysis compared with normal skin (Figure S8f,g, Supporting Information), demonstrating the good biocompatibility of the active DMN patch.

**Figure 4:**
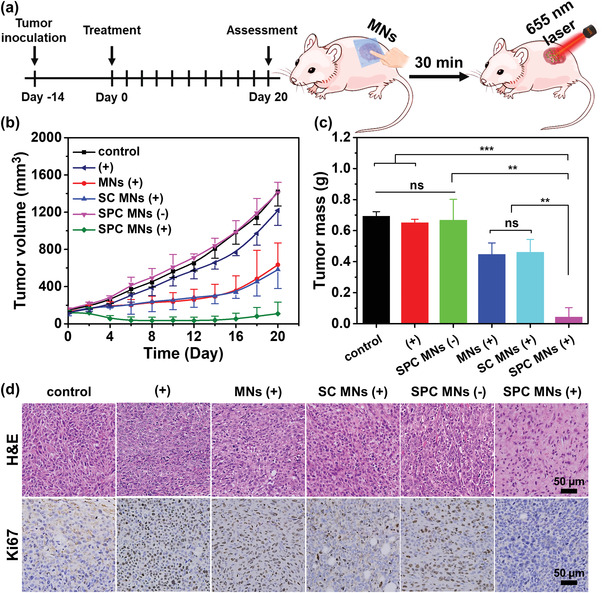
The enhanced PDT of Ce6‐loaded active DMNs in vivo. a) In vivo 4T1 tumor treatment by active DMNs. b) Tumor volume of each group within a 20‐d treatment (*n* ≥ 8), c) tumor mass (*n* = 4), d) H&E staining and Ki67 immunohistochemical staining (brown) of tumor in different groups. The scale bar in the last image applies to the others in (d). The results in (b) and (c) were shown as mean ± SD. Significance was assessed by two‐way ANOVA and Student's *T*‐test. ns, *p* > 0.05, *p < 0.05, **p < 0.01, ***p < 0.001.

To demonstrate the enhanced PDT by the Ce6‐loaded active DMNs, the intradermal breast cancer mouse model (4T1) was used to evaluate the in vivo PDT effect of the Ce6‐loaded DMNs. Specifically, 4T1 cells were cultured in the dermis of Balb/c mice, when the subcutaneous tumors grew to 5–6 mm in diameter (volume of ≈ 100 mm^3^), different treatment methods were adopted every other day: no treatment (control), laser (+), Ce6‐loaded SPC MNs (SPC MNs (−)), Ce6‐loaded MNs with laser irradiation (MNs (+)), Ce6‐loaded SC MNs with laser irradiation (SC MNs (+)), Ce6‐loaded SPC MNs with laser irradiation (SPC MNs (+)). The MN patches were inserted into the tumor on the right side of the mouse and kept for 5 min, followed by irradiation of 655 nm laser at 0.56 W cm^−2^ for 3 min after 30 min (**Figure** **4**a). Different administrations were applied every two days, and the tumor‐inhibited effect was evaluated by tumor growth, tumor mass, body weight, survival rate, and systematic biocompatibility. After a 20‐d treatment, the tumor volumes of experimental groups were lower than the control groups. As expected, PDT delayed tumor growth compared to untreated animals (Figure 4b). Besides, there was no difference between the laser group and the control group, suggesting that the low laser power density cannot kill the tumor cells at low temperature (Figures S9 and S10, Supporting Information). The alkaline solution had no obvious influence on tumor growth‐inhibited; therefore, SC MNs (+) group showed a similar tumor growth curve to the MNs (+) group. With the gaseous oxygen released from SPC MNs, the tumor growth rate was inhibited for the relief of hypoxia in the tumor transitorily (Figure S11, supporting information). Owing to the promoted penetration by gaseous oxygen released from SPC (Figure S12a,b, Supporting Information), the tumor volume of the SPC MNs (+) group was lower than other groups. Although ROS might have a killing effect on normal cells, most of the ROS will be preferentially consumed by more sensitive tumor cells as most Ce6 was present in the tumor. After the whole treatment period, the tumor mass of SPC MNs (+) (0.043 ± 0.061 g) and relative tumor volume were lower than that of control (0.693 ± 0.029 g) (Figure 4c; Figure S13a, Supporting Information), and the hematoxylin and eosin (H&E) staining and Ki67 immunohistochemical staining of the tumor showed that the lower cell density and slower tumor cells proliferation of SPC MNs (+) group, further confirmed the more effective PDT effect (Figure 4d). Body weight kept a slow increase during the treatment, suggesting that SPC MNs (+) treatment had no obvious side effects on the other organs (Figure S13b, Supporting Information). After the improved PDT treatment, the survival rate was prolonged, implying that the treatment of SPC MNs with laser irradiation could prolong the survival time of 4T1 tumor‐bearing mice (Figure S13c, Supporting Information). Furthermore, we assessed the biosafety through H&E stain of organs, the results showed that there was no damage to the other organs (Figure S14, Supporting Information). The above results show that Ce6‐loaded active DMNs can improve the PDT effect, and have lower system toxicity and good compatibility in vivo.

## 3 Conclusion

In summary, we demonstrated PS‐loaded DMN patches with O_2_ propellant for enhanced transdermal delivery of PS and improved PDT by embedding SPC microparticles into dissolving PVP MN matrix. Once the MNs were inserted into the skin, the embedded SPC microparticles were exposed to the interstitial fluid and generated gaseous oxygen bubbles to form a violent vortex for promoting the migration of PS. Moreover, the generated oxygen could relieve the hypoxia in 4T1 tumors during the PDT process, increasing the antitumor effect with fewer side effects. This strategy provides a powerful PDT platform especially for the treatment of tumors, which also can be extended to a wide range of therapeutic applications for enhanced outcomes.

## 4 Experimental Section

### Materials

Polydimethylsiloxane (PDMS, Sylgard184) was purchased from Dow Corning Corp. (USA). PVP (*M*_w_ = 58 kDa) and sodium percarbonate (SPC) were bought from Aladdin Biochemical Technology Co., Ltd (Shanghai, China). Sodium carbonate (SC), dimethyl sulfoxide (DMSO, AR), and agarose (BR) were provided by Sinopharm Chemical Reagent Co., Ltd (Beijing, China). Chlorins e6 (Ce6) was purchased from Frontier. F‐127 was purchased from Sigma (USA). 2,7‐Dichlorofluorescin diacetate (DCFH‐DA) was purchased from Beyotime Biotechnology Co., Ltd (China). Calcein‐AM/PI live/dead cell staining kit was provided by Yeasen Biotechnology Co., Ltd (Shanghai, China).

### Preparation of Ce6‐Loaded MNs

MNs were fabricated by a two‐step process by a micro‐molding method. Briefly, a mixture of SPC (300 mg mL^−1^), PVP (400 mg mL^−1^), and Ce6 (1 mg mL^−1^) in DMSO was filled into the PDMS female mold under vacuum at a pressure of −0.08 MPa for 10 min, and the excess solution was removed. The mixture with the mold was then dried at 50 °C for 8 h. Subsequently, PVP solution in DMSO solution (500 mg mL^−1^) was added to the above PDMS mold under vacuum at a pressure of −0.05 MPa for 5 min to form a base, and then the sample was dried in an oven at 50 °C overnight, followed by being peeled off from the mold and stored in a desiccator at room temperature. The preparation of SC MNs was similar to that of SPC MNs with SPC being replaced by SC microparticles.

### MNs Characterization

Morphologies of MNs were observed by a digital microscope (AM4113ZTL, Dino‐Lite, Taiwan, China) and a scanning electron microscope (Nova NanoSEM 450, FEI, The Netherlands). The energy‐dispersive X‐ray spectroscopy (EDX) mapping analysis was carried out using a voltage of 18 kV. Bright‐field and fluorescent microscopy images of MNs were obtained by Olympus fluorescence microscope with a fluorescence filter for a blue‐light excitation. The gaseous oxygen generated during the dissolving process of active DMNs was tested by an oxygen dissolution tester (Mettler Toledo, Shanghai). First, eight active DMN patches were put into a 50 mL centrifuge tube containing 15 mL PBS solution (0.1 mol L^−1^, pH = 7.4). Then, the detector was put into the PBS. Taking the dissolving oxygen of PBS as a contrast, the generated oxygen of the active DMNs is obtained by the increment of the dissolving oxygen.

### Ce6‐Loaded SPC MNs Promoted Payload Diffusion

An aqueous solution of F‐127 (30 wt%) was weighed in a 50 mL centrifuge tube in phosphate buffer solution (0.1 mol L^−1^, pH 7.4) and stored at 4 °C until the solution became transparent. The solution was transferred to a plastic cuvette and cured at room temperature to obtain the F‐127 gel. Then, the Ce6‐loaded SPC MNs were inserted into the F‐127 gel, and the evolution of the red fluorescence signal was recorded by a microscope.

### Mechanical Property Test

The mechanical properties of the MN array were evaluated by the IBTC‐300 mechanical test system (Care‐MC) with a constant loading speed of 4 µm s^−1^. When the plate was in immediate contact with the MN tip, the mechanical strength was measured. The breaking point was determined when the force dropped sharply.

### COMSOL Multiphysics Simulation

A transient laminar flow model was used in COMSOL to simulate the flow process of payload in water. First, a 2D geometry was constructed and unstructured triangular meshes were created. The size of the meshes was controlled by physical field and there were 6318 meshes in total. Since the flow was multiphase, the Bubbly Flow (bf) interface was used to simulate the influence of bubbles on the velocity field, and employed the Transport of Diluted Species in Fractures (dsf) interface to simulate the convection and diffusion of payload. The coefficient of diffusion of the payload in water was set as 4.56×10^−10^ m^2^ s^−1^. Next, the boundary conditions were set: where the concentration of the inflowing payload at each capsule was 5.77×10^−5^ mol m^−3^, and the flow rate of gas was 2×10^−4^ kg (m^2^ s)^−1^. To simplify the geometry, the diffusion process was simulated of the three capsules, thus the boundaries were set periodically, and the top of the calculation region was set as the gas outlet condition. Finally, the transient flow process with a total duration of 120 s and time step of 0.1 s was calculated.

### Cellular ROS Detection

The 4T1 cells were cultured in 12‐well plates for 8 h and transferred to an anaerobic bag at 37 °C for 4 h, and were divided into 6 groups: (1) control, (2) laser, (3) Ce6‐loaded MNs + laser, (4) Ce6‐loaded SC MNs + laser, (5) Ce6‐loaded SPC MNs + laser, (6) SPC MNs + laser. A piece of 2 mm agarose gel was embedded into each well and added the DCFH‐DA probe for 30 min incubation, then treated it with MNs and sealed it in the anaerobic bag (1% O_2_) at 37 °C for another 30 min. After removing the gel, the wells were irradiated with 655 nm laser with an intensity of 0.1 W cm^−2^ for 3 min and observed under the fluorescent microscope. The average fluorescence intensity (FL intensity) was calculated using Image J by the equation: average FL intensity = (Integrated Density)/area.^[^ ^21^ ^]^

### Cell Viability

To evaluate the cell‐killing ability of Ce6‐loaded SPC active MNs under 655 nm laser, the groups were divided as: (1) control, (2) laser, (3) Ce6‐loaded MNs + laser, (4) Ce6‐loaded SC MNs + laser, (5) Ce6‐loaded SPC MNs + laser, and (6) SPC MNs + laser. The 4T1 cell monolayers were cultured in 24‐well plates for 8 h and transferred into an anaerobic bag for 4 h to create a hypoxic atmosphere. The 2 mm agarose gel was tiled above the monolayers before punctuating the MNs. Then, each well was sealed and the plate was kept in the anaerobic bag (1% O_2_) for another 30 min, and the wells were illuminated with 655 nm laser at 0.1 W cm^−2^ for 3 min after the removal of agarose gel. Lastly, the cell viability was tested by CCK8 according to the user's guide.

### Live/dead Cell Staining

The 4T1 cells were plated in 12‐well plates and the wells were put into an anaerobic bag for 4 h, followed by being classified as: (1) control, (2) laser, (3) Ce6‐loaded MNs + laser, (4) Ce6‐loaded SC MNs + laser, (5) Ce6‐loaded SPC MNs + laser, (6) SPC MNs + laser, and were put into an anaerobic bag (1% O_2_) for 4 h before PDT treatment (0.1 W cm^−2^, 3 min). After the treatment, the cells were placed at 37 °C for a 4 h‐incubation. The Calcein‐AM/PI staining was performed according to the manufacture's instructions. Cells stained green (alive) and red (dead) were imaged under the fluorescence microscope.

Animals

Female Balb/c mice (18–20 g) were purchased (Changsheng Biological Technology Co., Ltd, Liaoning, China) and were housed in a temperature‐controlled room (22 ± 1 °C) on a 12 h light‐dark cycle with free access to food and water, and raised in the specific pathogen‐free condition. All animal experiments were carried out in accordance with the Guide for the Care and Use of Laboratory Animals of Huazhong University of Science and Technology, approved by the Institutional Animal Care and Use Committee, Tongji Medical College, Huazhong University of Science and Technology (IACUC Number: 2680).

### SPC Active MNs Improved PDT In Vivo

The 4T1 tumor‐bearing mice were established by intradermal injection of 1×10^6^ cells per mouse on the right buttocks. The PDT treatment was started when the tumor reached 100 mm^3^. The mice were randomly divided into 6 groups (8–10 mice/group): (1) control, (2) laser, (3) Ce6‐loaded MNs + laser, (4) Ce6‐loaded SC MNs + laser, (5) Ce6‐loaded SPC MNs, and (6) Ce6‐loaded SPC MNs + laser. The tumors were treated by MNs for 5 min to allow the MNs to dissolve completely, and the tumor was irradiated with 655 nm laser (0.56 W cm^−2^) for 3 min after 0.5 h. All the treatments were given every other day. The other group without treatment was employed as a control. The volume of the tumor was monitored by a caliper every other day and calculated by the following equation: *V* = *L*_tumor length_ × (*W*_tumor width_)^2^/2. Simultaneously, the weight of the tumor was recorded. After treatment was completed, the mice were sacrificed, and then the tumor and major organs were dissected for further H&E staining and Ki67 immunohistochemical staining.

### Statistical Analysis

All data are obtained from at least three independent experiments with three or more parallel samples per condition in each experiment and are expressed as mean ± standard deviations (SD). Significance was assessed with two‐way ANOVA and Student's *T*‐test. The probability value of *p < 0.05, **p < 0.01, ***p < 0.001, ****p < 0.0001 was considered significant. GraphPad Prism 7 was used for statistical analysis.

## Conflict of Interest

The authors declare no conflict of interest.

## Supporting information

Supporting Information

Supplemental Video 1

Supplemental Video 2

## Data Availability

The data that support the findings of this study are available from the corresponding author upon reasonable request.
