# Supplementary material for: Microneedle Patches with O2 Propellant for Deeply and Fast Delivering Photosensitizers: Towards Improved Photodynamic Therapy
Source: Adv Sci (Weinh). 2022 Jul 15;9(25):2202591. doi: 10.1002/advs.202202591 (PMC9443460; doi:10.1002/advs.202202591)
Supplement: Supplementary file 1 — Supporting Information [file ADVS-9-2202591-s001.pdf]

ADVANCED SCIENCE
Open Access

Supporting Information

for *Adv. Sci.*, DOI 10.1002/advs.202202591

Microneedle Patches with O_2 Propellant for Deeply and Fast Delivering Photosensitizers: Towards Improved Photodynamic Therapy

Pei Liu, Yangxue Fu, Fulong Wei, Teng Ma, Jingli Ren, Zhanjun Xie, Shanzheng Wang, Jinjin Zhu, Lianbin Zhang, Juan Tao* and Jintao Zhu**

Supporting Information for:

Microneedle Patches with O_2 Propellant for Deeply and Fast Delivering Photosensitizers: Towards Improved Photodynamic Therapy

Pei Liu, Yangxue Fu, Fulong Wei, Teng Ma, Jingli Ren, Zhanjun Xie, Shanzheng Wang, Jinjin Zhu, Lianbin Zhang, Juan Tao,* and Jintao Zhu**

P. Liu, T. Ma, Dr. J. Ren, Z. Xie, S. Wang, Prof. L. Zhang, Prof. J. Zhu
Hubei Engineering Research Center for Biomaterials and Medical Protective Materials, and State Key Laboratory of Materials Processing and Mold Technology, School of Chemistry and Chemical Engineering, Huazhong University of Science and Technology (HUST), Wuhan 430074, China
*E-mail: zhanglianbin@hust.edu.cn (L. Z.); jtzhu@mail.hust.edu.cn (J. Z.)

Y. Fu, Dr. J. Zhu, Prof. J. Tao
Department of Dermatology, Union Hospital, Tongji Medical College, HUST, Wuhan 430022, China
*E-mail: tjhappy@126.com (J. T.)

F. Wei
School of Energy and Power Engineering, HUST, Wuhan 430074, China

Supporting Figures:

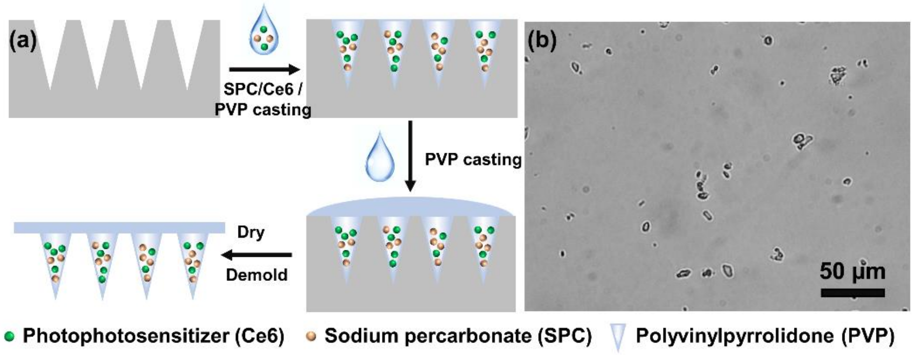

Figure S1. (a) Illustration showing the fabrication process of Ce6-loaded active DMNs and (b) optical microscopy image of the SPC microparticles.

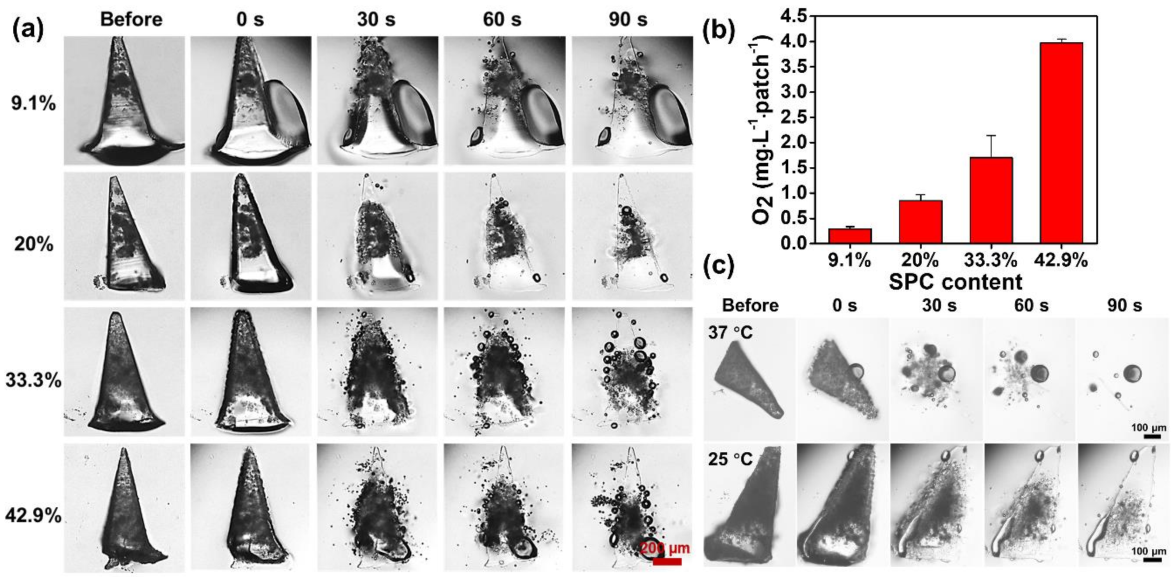

Figure S2. Characterization of O_2 generated by active DMNs. (a) Time-course optical microscopy images of a single MN made of different feeding SPC contents in PBS solution. Scale bar in the last image applies to the others. (b) Concentration of the gaseous oxygen in PBS generated by one MN patch made of different feeding SPC contents (n = 3). (c) Time-course optical microscopy images of a single MN needle with feeding SPC contents of 42.9% in PBS solution at 25 ℃ and 37 ℃, respectively. At 90 s, the MN dissolved completely at 37 ℃, while ~10% of it remained at 25 ℃, indicating the dissolving of polymer MNs and O_2 generation was accelerated as the temperature increased.

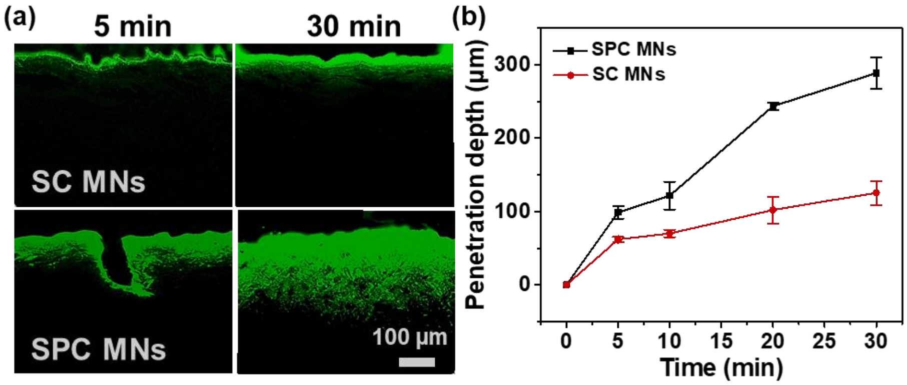

Figure S3. Cross-sectional fluorescence microscopy images (a) and penetration depth (b) of fluorescein isothiocyanate isomer I (5-FITC) by MNs at different time points in pigskin. Scale bar in the last image applies to the others in (a). The results showed the distribution of green fluorescence signals released from MNs in the cross-sectional images using the pigskin as a model. Limited by the shape and imperfect insertion of the MNs, the payload will be preferentially deposited on the skin surface after being inserted into the skin. Compared with the delayed delivery of passive SC MNs, the embedded SPC particles greatly enhanced the delivery of 5-FITC. At 30 min, the average penetration depth of passive SC MNs was 125 ± 16 µm, while the value observed in active SPC MNs was 289 ± 22 µm (n = 3), this improved permeability and the faster transport were significantly pronounced.

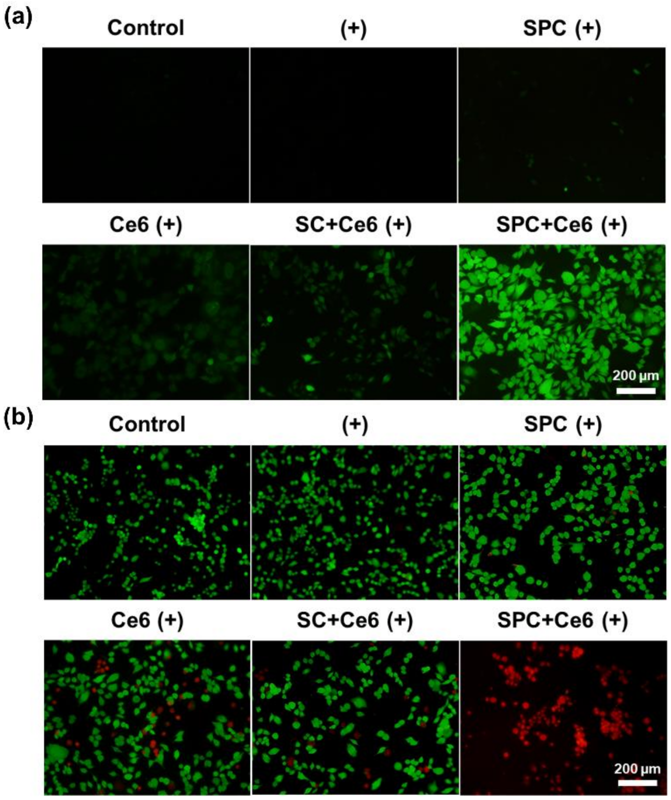

Figure S4. *In vitro* PDT activity of SPC aqueous suspension with 1 μg/mL Ce6. (a) Fluorescence microscopy images of hypoxic 4T1 cells after being treated with PBS (control), PBS+laser (+), SPC solution, Ce6 solution, SC/Ce6 solution, and SPC/Ce6 solution, followed by laser irradiation (655 nm, 0.1 W/cm^2, 3 min), and stained by DCFH-DA probe. (b) Calcein-AM/PI staining of 4T1 cells after treating with PBS (control), PBS+laser (+), SPC solution, Ce6 solution, SC/Ce6 solution, and SPC/Ce6 solution, followed by laser irradiation. The scale bar in the last image applies to the others in (a) and (b).

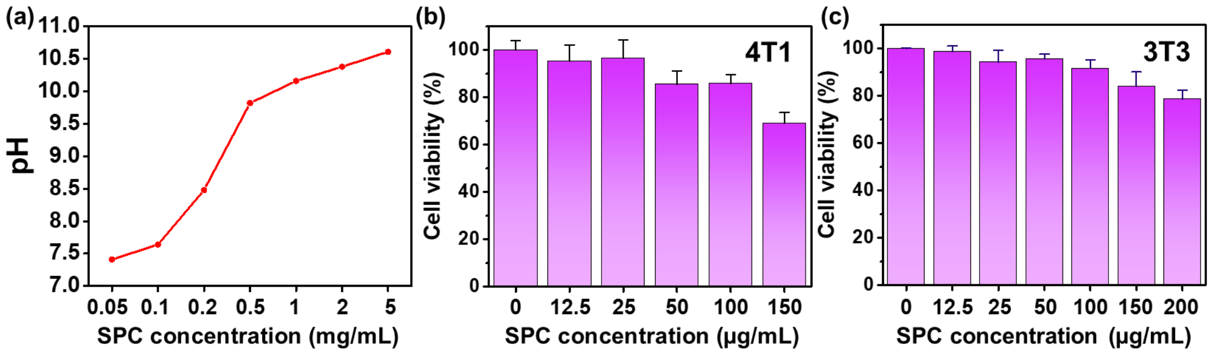

Figure S5. (a) Influence of SPC concentration on pH of 10 mL PBS solution (0.1 mol/L, pH 7.4). Cell viability of hypoxic 4T1 cells (b) and 3T3 cells (c) after being incubated with different concentrations of SPC suspensions ($n \geq 3$).

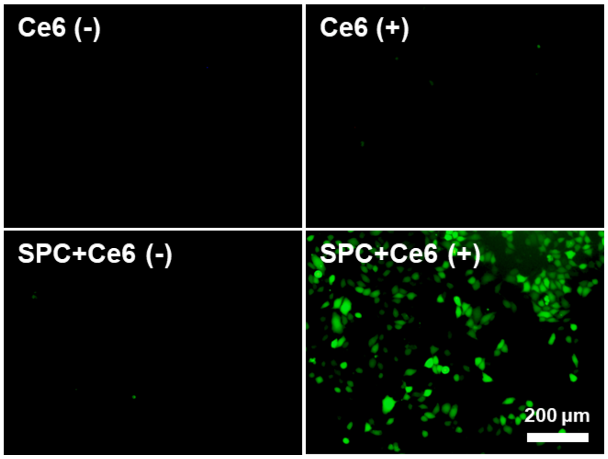

Figure S6. Fluorescence microscopy images of hypoxic 4T1 cells (1% O_2) treated with Ce6-loaded MNs with/without laser irradiation and stained by DCFH-DA probe. Scale bar in the last image applies to the others. While without laser irradiation, there was no change in fluorescence signal level, indicating that the weak oxidation property of SPC aqueous solution did not affect the probe. While with laser irradiation, the fluorescence signal level in 4T1 cells treated with Ce6-loaded SPC MNs increased significantly compared with that of cells treated with Ce6-loaded MNs, indicating that the SPC could promote the downward migration of the Ce6 and improve the hypoxic microenvironment of 4T1 cells.

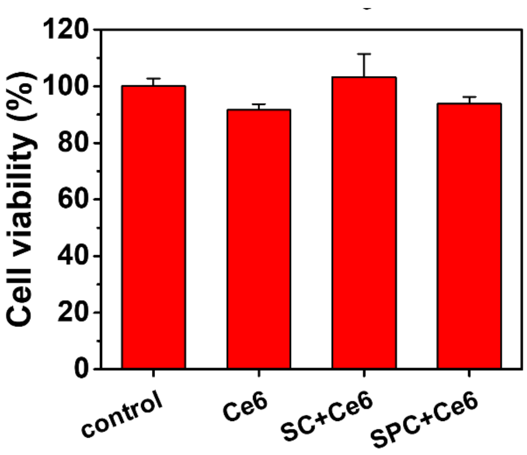

Figure S7. Viability of hypoxic 4T1 cells (1% O_2) after being incubated with Ce6-loaded MNs for 24 h ($n \geq 3$).

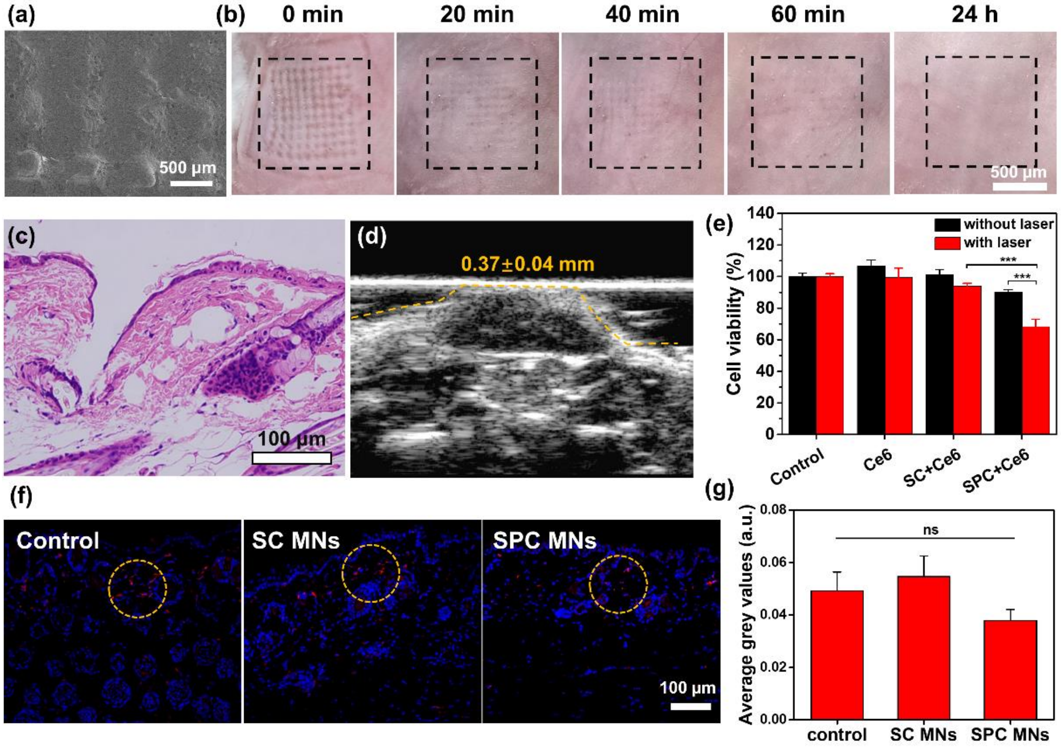

Figure S8. The insertion and compatibility of active DMNs. (a) SEM image of Ce6-loaded active DMNs after being inserted into mice dorsal skin for 5 min. (b) Optical microscopy images of the hair removed dorsal mice skin after the removal of active DMNs. (c) The image of H&E-stained mouse skin after treatment with active DMNs for 5 min. Due to the high elasticity of the skin and limited by the operation, the final puncture depth (~ 200 μm) in the histological section was much less than the height of MN. (d) Typical skin ultrasound image of the tumor site. (e) Cell viability of 3T3 cells after being incubated with Ce6-loaded MNs for 24 h with/without laser irradiation (1 patch/mL). The cell viability of 3T3 cells remained > 90% after being treated with different groups, indicating that the MNs had good biocompatibility. IL-1β (red) immunohistochemical staining (f) and corresponding statistical analysis (g) in mice dorsal skin 24 h after MNs insertion. Scale bar in the last image applies to the others in the same panel of (a) and (c). The results in (e) and (g) were shown as the mean ± SD. Statistical significance was calculated by student's *T*-test ($n \geq 3$). ns, $p > 0.05$, *$p < 0.05$, **$p < 0.01$, ***$p < 0.001$.

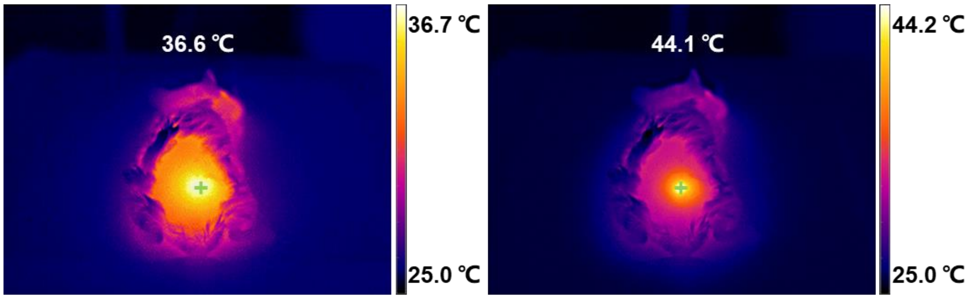

Figure S9. Thermal images of Ce6-loaded active DMNs-treatment mice before (left) and after (right) 655 nm laser irradiation at 0.56 W/cm^2 for 3 min.

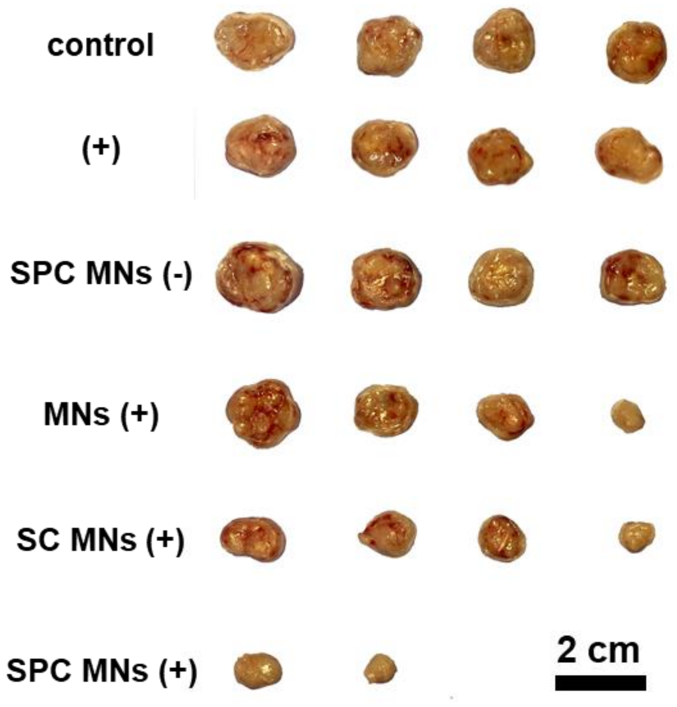

Figure S10. Photographs of the tumors of each group at day 20th (n = 4).

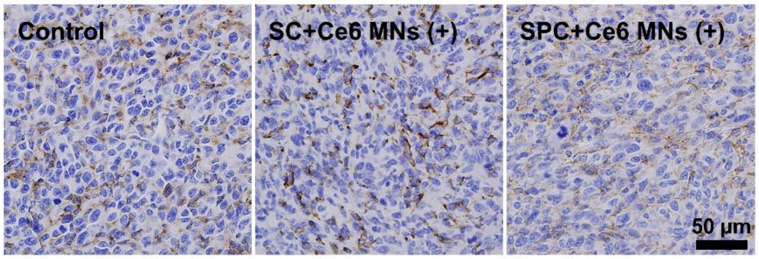

Figure S11. HIF-1α staining (hypoxia inducible factor-1, brown) analysis of tumor tissues at 24 h after different treatments. The scale bar in the lower-right image applies to the others. The tumor was treated by Ce6-loaded MNs and dissected for further histological sections and HIF-1α staining. 24 h later, the amount of HIF-1α decreased after being treated with Ce6-loaded SPC MNs compared to the control and Ce6-loaded SC MNs treatment, indicating that the improvement of hypoxia of tumor by the O_2 propellant-loaded MNs.

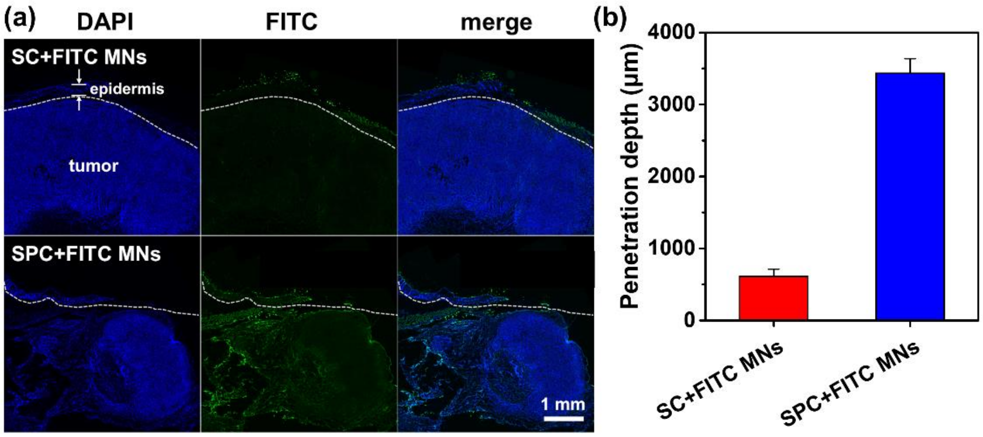

Figure S12. (a, b) The penetration and distributions of 5-FITC in tumor-bearing mice at 0.5 h after 5-FITC-loaded MNs (including SC MNs and SPC MNs) treatments: (a) Fluorescence microscopy images of histological sections of the tumor (blue signal: DAPI; green signal: 5-FITC); (b) the penetration depth of 5-FITC. Model molecule (5-FITC)-loaded MNs were inserted into the breast tumor-bearing mice for 5 mins until the MNs completely dissolved. 0.5 h later, the tumor with skin was dissected for histological section and further fluorescence staining (DAPI). With the aid of O_2, most of the 5-FITC (green fluorescence signal) traveled through the epidermis and reached the lower middle tumor (SPC MNs: 3436 ± 203 μm vs SC MNs: 613 ± 100 μm). Solubility was one of the most important factors affecting molecule diffusion. Therefore, we can infer the penetration of hydrophobic small-molecular of Ce6 was similar to that of 5-FITC. Similarly, we can infer that most Ce6 could reach the deep tumor. The results in (b) were shown as mean ± SD (n = 3). ns, $p > 0.05$, *$p < 0.05$, **$p < 0.01$, ***$p < 0.001$.

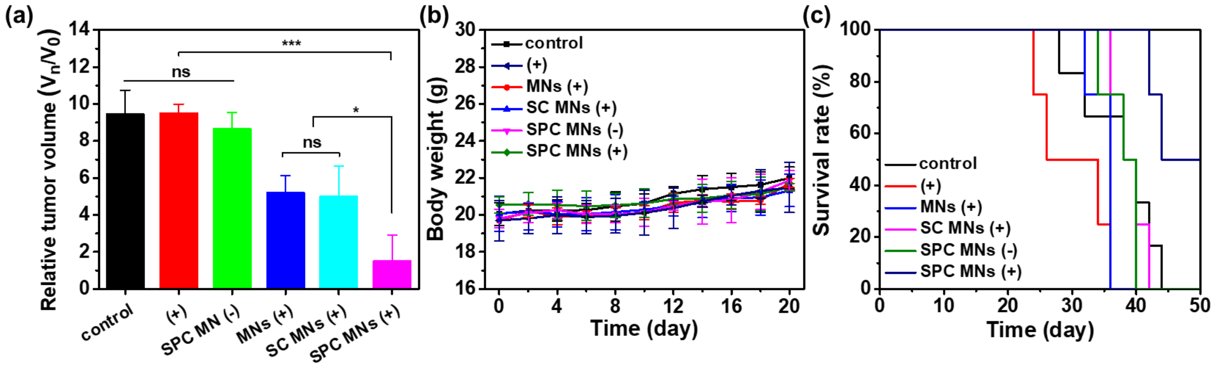

Figure S13. The PDT of Ce6-loaded active DMNs *in vivo*. (a) Relative tumor volume of each group at day 20th (n ≥ 8); (b) body weight within 20-day (n ≥ 8), (c) survival rate within the periods of observation (n ≥ 4). The results in (a-c) were shown as the mean ± SD. Statistical significance was calculated by student's *T*-test. ns, $p > 0.05$, *$p < 0.05$, **$p < 0.01$, ***$p < 0.001$.

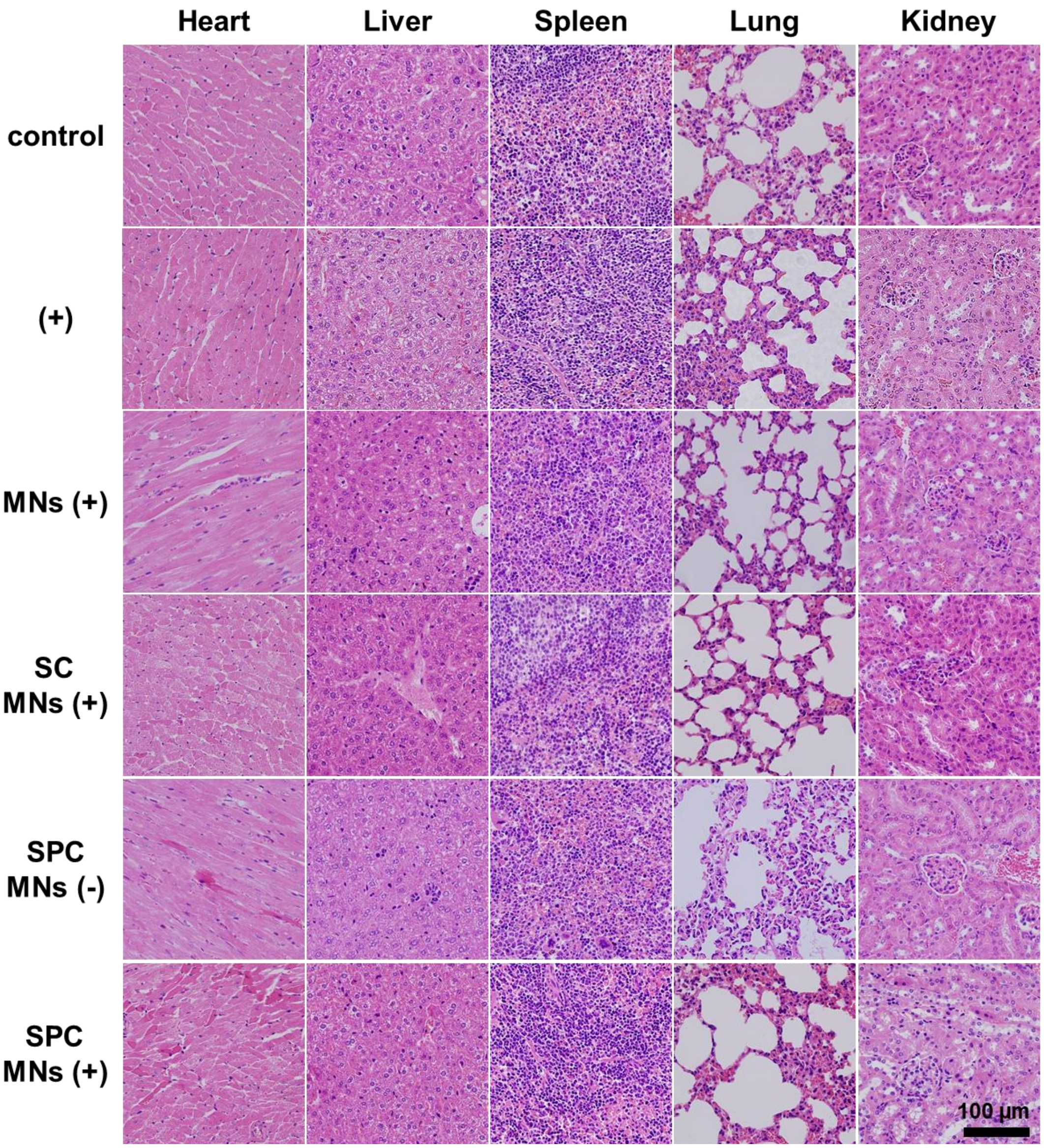

Figure S14. Histological examination of major organs (heart, liver, spleen, lung, and kidney) sections stained by H&E from 4T1-bearing mice after different treatments. Scale bar in the last image applies to the others.

Supplementary Videos:

Captions for the Supplementary Movies

Supplementary Video S1. Real time video shows the dissolution state of Ce6-loaded active SPC MNs in PBS.

Supplementary Video S2. Real time video shows the dissolution state of Ce6-loaded passive SC MNs in PBS.
